# The Impact of Newly Diagnosed Early Breast Cancer on Psychological Resilience, Distress Levels, and the Perception of Health

**DOI:** 10.3390/ijerph21060677

**Published:** 2024-05-24

**Authors:** Anuska Budisavljevic, Natalija Dedic Plavetic, Kristina Klaric, Renata Kelemenic-Drazin, Marina Letica-Crepulja

**Affiliations:** 1 Department of Medical Oncology and Hematology, General Hospital Pula, 52100 Pula, Croatia; 2 Department of Oncology, University Hospital Centre Zagreb, School of Medicine, University of Zagreb, 10000 Zagreb, Croatia; ndedic@kbc-zagreb.hr; 3 Department of Radiology, General Hospital Pula, 52100 Pula, Croatia; klarickristina8@gmail.com; 4 Department of Hematology, Oncology and Clinical Immunology, General Hospital Varazdin, 42000 Varazdin, Croatia; renata.kelemenic-drazin@obv.hr; 5 Department of Psychiatry and Psychological Medicine, Faculty of Medicine, University of Rijeka, 51000 Rijeka, Croatia; marinalc@medri.uniri.hr

**Keywords:** psychological resilience, breast cancer, psychological distress, stressors, quality of life, psychological well-being, mental health, psychological disorders

## Abstract

Confronting a breast cancer diagnosis, along with complex and challenging treatment procedures, is an extremely stressful experience. Psychological resilience is the ability to maintain or restore normal functioning while facing adversity. We aimed to explore the impact of an early breast cancer diagnosis on psychological resilience, distress, and perception of health. A cross-sectional study was conducted, including 50 patients newly diagnosed with early breast cancer and 67 healthy women with screening mammograms graded 1 or 2 using a Breast Imaging Reporting and Data System. The levels of distress, perception of health, and psychological resilience were assessed using the depression, anxiety, and stress scale, the SF 36-Item Health Survey 1.0, and the Connor–Davidson RISC-25 scale. Differences between variables were examined using the *t*-test and chi-square test for interval and categorial variables. The surveys were conducted within four weeks of a breast cancer diagnosis. Patients with breast cancer reported a deterioration of their health relative to the previous year and significantly higher levels of psychological resilience, while there was no significant difference between the groups in levels of stress, anxiety, or depression. The process of diagnosis with early breast cancer may activate psychological dynamic processes which are involved in the effective adaptation to acute stress, leading to higher resilience levels in breast cancer patients compared to healthy controls.

## 1. Introduction

Breast cancer (BC) is the most common cancer in women, with 2.3 million new cases diagnosed annually [1], around 15% of which are metastatic and the remainder are early BC (EBC is when the disease confined to breast and regional lymph nodes) [2]. Depending on the quality of screening programs and other treatment-related resources, the likelihood of five-year survival rates for patients with EBC can reach 96% [3].

Surgery is still the cornerstone treatment for EBC. Different EBC subtypes are additionally treated with systemic anti-cancer therapy such as chemotherapy, anti HER2 targeted therapy, immunotherapy, or anti-hormonal therapy. Locoregional radiotherapy is intended for women with breast-conserving surgery and/or positive axillary lymph nodes. Both systemic anti-cancer therapy and locoregional radiotherapy are delivered with the aim of reducing the risk of local and distant BC recurrence [3].

EBC-related treatments are known to be associated with physical, functional, psychological, cognitive, and economic side effects, impairing the quality of life (QoL) in BC patients [4,5,6]. On the whole, EBC as a potentially life-threatening disease, alongside complex and challenging treatment procedures, can be defined as an adversity with a profound effect on the physical and psychological well-being of those patients [7,8].

Psychological resilience is the ability to maintain or restore normal functioning while facing adversity, or the ability to “bounce back” from a stressful event [9]. Though widely researched, psychological resilience is a construct that still lacks a definition, and whether it should be defined as a trait or a dynamic process is still debated [10,11,12,13]. Epidemiological studies showed that the vast majority of the population had experienced at least one violent, tragic, or extremely stressful event in their lifetime, but not all developed stress-related psychological disorders [9,14], suggesting that psychological adaptation through resilience could play a protective role [11]. Subsequently, resilience has been defined, not only as a rare trait of exceptional individuals but rather as a dynamic process of the effective adaptation to life adversities, which occurs relatively frequently [10,11,15,16]. Furthermore, findings suggest that moderate lifetime exposure to adverse events helps build resilience and so enables ones capacity for the effective adaptation to future stressors, a process known as the “vaccination” effect [17,18,19,20].

Regarding patients with cancer, including BC patients, resilience refers to an individual’s protective mechanisms and personal characteristics which are amendable and can promote the successful adaptation to a cancer diagnosis [8]. Higher resilience levels are found to be associated with lower levels of distress, better QoL, better tolerance of treatment side effects and cancer-related fatigue [21,22,23,24,25,26], better body image, less depression and anxiety, and less severe adverse effects of systemic anti-cancer treatment [27,28]. Regarding sociodemographic characteristics, male sex, older age, and a higher education level are positively related to resilience in some studies while inversely associated in others [8,20,21,23]. On the other hand, social support, higher income, time since diagnosis, adjuvant chemotherapy, and trust in treatment enhance resilience in patients with cancer [8,29,30,31].

Considering women with BC, the literature review shows that they have the ability to resist and accept cancer adversity [31,32,33,34]. Still, studies on resilience in BC patients had a wide heterogeneity of the BC patients. While some studies assessed the resilience of EBC patients during active oncology treatment [25,26,27,29,35], others assessed resilience among BC survivors [17,28,31,32,35,36], and few combined early and metastatic BC patients [15,16,24,37]. Furthermore, very few studies were designed with a control group [29,32,33,35,38], or included a BC group that consisted of early and metastatic BC patients. Taking into account potential confounding factors of the BC treatment trajectory and survivorship, it was difficult to conclude what the direct impact of BC diagnosis on resilience levels is.

The aim of this study was to explore whether being diagnosed with EBC has an impact on the resilience levels assessed before starting the treatment, and if it does, would it enhance or decrease resilience. Additionally, we wanted to explore the impact of an EBC diagnosis on distress and the perception of health.

## 2. Materials and Methods

### 2.1. Study Design

This cross-sectional study was conducted in General Hospital Pula. In order to examine the impact of a EBC diagnosis on psychological resilience, distress, and perception of health, we formed two groups: the early breast cancer (EBC) group of women who have just received the breast cancer diagnosis and the control group of healthy women. Upon obtaining approval from the Institutional Ethics Committee Board, data were collected at the Oncology Outpatient Clinic from December 2021 until September 2022. Patients diagnosed with stage I–III EBC [39] were screened to participate in the study at the first oncology appointment after they had been informed about the BC diagnosis and the treatment plan had been discussed. Patients with in situ breast cancer were not included in the study. The surveys were completed before the start of active oncology treatment within four weeks of the screening because we wanted to exclude the impact of EBC treatment. The control group was recruited from women who applied for screening mammography through the Regional and National Breast Cancer Screening Programs, and whose screening mammography findings were normal, namely, BI-RADS 1 and BI-RADS 2, according to Breast Imaging Reporting and Data System (BI-RADS) [40]. The healthy women were offered to participate in the study when they were handed the results of the mammography screening at Radiology Department.

The Regional and National Breast Cancer Screening Programs in Croatia are designed for women between 40 and 70 years of age, so the age range for our control group was between 40 and 70 years. For the EBC group, the age range of participants was allowed to be 18–70 years, since only 5% of EBC patients are diagnosed under the age of 40 [41,42]. The exclusion criteria for both groups were as follows: psychiatric conditions currently or previously requiring medication, previously defined cognitive impairment, and cancer other than EBC that required active oncology treatment. After providing informed consent, participants were instructed to complete the self-reported surveys alone; the surveys were conducted at an outpatient clinic both for EBC patients and healthy control. In cases of difficulties with understanding questions, a trained data collector helped with reading, understanding, and completing the questionnaires.

### 2.2. Measurement Instruments

Basic demographic data on respondent age, marital status, dependent children, education, employment, income, comorbidities, and medical history were collected.

Due to the fact that former adverse experiences could have an impact on resilience [17,18], assessment of previous lifetime exposure to traumatic and threatening experiences was made for both groups. Previous lifetime exposure to traumatic events was assessed using two scales: The Life Events Checklist DSM-5 (LEC) [43] and The List of Threatening Experiences (LTE) [44]. LEC is a self-reported scale that assesses exposure to 16 events known to potentially result in post-traumatic distress syndromes or distress, such as exposure to war, domestic violence, or natural disasters. LTE checklist is composed of 12 threatening experiences related to personal problems, spousal and relational problems, employment and financial problems, illness, and bereavement. Participants were asked two questions. Firstly, whether they have ever experienced any traumatic event listed on the LEC checklist, and secondly, whether they have experienced any threatening event in the last 6 months according to the LTE checklist.

The Depression Anxiety Stress Scale (DASS-21) was used to measure levels of distress [45]. DASS-21 is a set of three self-report scales designed to measure the emotional states of depression, anxiety, and stress. It consists of 21 items, 7 items of which are for each dimension and participants answer referring to the past week on a 4-point Linkert scale ranging from 0 (“Did not apply to me at all”) to 3 (“Applied to me very much or most of the time”). The DASS-21 scale is widely used in clinical settings and research and validated in the Croatian population [46]. In this study, Cronbach’s α coefficients of reliability were satisfactory: 0.909 for stress, 0.857 anxiety, and 0.861 for depression.

The SF 36-Item Health Survey 1.0 (SF-36) self-reported scale was used to evaluate health perception [47]. It consists of 35 items which assess two domains, physical and emotional health. Each domain has four subscales: physical health including physical functioning, bodily pain, role limitations due to physical health problems, and role limitations due to personal or emotional problems and emotional health including general mental health, social functioning, energy/fatigue, and general health perception. The SF-36 scale also includes one additional item to evaluate perceived change in general health compared to 1 year prior. Higher scores indicate better functioning. The SF-36 was validated in the Croatian population [48]. In this study, the Cronbach’s α coefficients were as follows: 0.897 for physical functioning, 0.862 role limitations due to physical health, 0.783 role limitations due to emotional problems, 0.812 energy/fatigue, 0.884 emotional well-being, 0.735 social functioning, 0.880 pain, and 0.619 general health.

Psychological resilience was assessed using the self-reported Connor–Davidson Resilience Scale, (CD-RISK 25) [49], which has one dimension. The scale comprises 25 questions. Participants answer the questions referring to the past month on a 5-point Linkert scale, ranging from 0 (“Not true at all”) to 4 (“True nearly all the time”). Higher scores indicate greater resilience levels. Questions are related to different aspects of resilience over the past month, including hardiness, coping, adaptation/flexibility, meaningfulness/purpose, optimism, regulation of emotion, and cognition and self-efficacy. CD-RISC-25 has been tested in the general population and different clinical samples and excellent psychometric properties were shown in both cases. It has been used to measure outcomes of interventions fostering resilience, as scores can change over time during treatment/counseling/stress management, to reflect resilience growth [38,50,51]. Permission to use an approved Croatian translation of the scale by Jaksic & Jakovljevic was obtained from the authors. Internal consistency of the scale was high, with Cronbach’s Alpha 0.901.

### 2.3. Statistical Analysis

Statistical analyses were conducted using IBM SPSS Statistics for Windows version 26.0. Descriptive data are presented as means, standard deviations (SD), and percentages. Differences between variables were examined using the *t*-test and chi-square test, for interval and categorial variables, respectively, setting 5% as constituting a significant difference. We made adjustment for age by using age as covariate. The required sample size was calculated using Gpower, based on previous studies [29,35,36]. A minimum of 27 subjects per group was calculated as required for study power 0.95 and significance level *p* < 0.05.

## 3. Results

There were 56 women screened for inclusion in the EBC group, 4 of which refused to participate, (response rate 92%) 1 was diagnosed with metastatic BC, and 1 was diagnosed with another primary tumor; therefore, the final EBC group comprised 50 participants. A total of 130 women were approached to participate as controls, and 68 of them agreed (a response rate of 52%), 1 of whom was excluded because of a lung carcinoma; thus, the final control group comprised 67 participants. Participant demographic characteristics are presented in Table 1.

Participant age ranges were 27–70 (mean = 57.94, SD = 10.24) years and 40–70 (mean = 57.01, SD = 9.62) years in the EBC and control groups, respectively (t = −0.501, df = 115, *p* = 0.618). The groups were well balanced considering all the demographic characteristics. According to the LEC almost half of the participants in both groups (EBC, 48.0%; control, 44.8%) had experienced a previous traumatic event, while around one-third (EBC 26.0%; control 31.3%) had experienced a threatening event in the last 6 months, according to the LTE.

The results of the DASS-21, SF-36, and CD-RISC-25 scales are shown in Table 2. The EBC group had significantly higher CD-RISC-25 scores than the control group, indicating higher levels of psychological resilience (t(df) 2.530, *p* < 0.05). Also, patients in the EBC group had significantly lower scores on the single SF-36 item evaluating perceived change in general health compared to 1 year prior than those in the control group (t (df) 3.835, *p* < 0.01)**,** while no significant differences in the four domains of physical and emotional health perception were detected between the EBC and control group. Furthermore, there were no significant differences between the EBC and control groups in any DASS-21 subscale. Most participants in both groups had no symptoms of depression (EBC 66%; controls 82%) or anxiety (EBC 78%; controls 89%). Severe depression was noted in 10% and 3%, severe anxiety in 8% and 4.5%, and severe stress in 10% and 4% of patients with EBC and controls, respectively.

## 4. Discussion

In this study, EBC group participants had higher resilience levels and reported a worse perception of health relative to controls. Also, we found a greater prevalence of severe depression, anxiety, and stress when comparing the EBC and control groups but without statistical significance, which could be driven by a small sample size.

Systematic reviews have reported prevalence rates of 9.4–66.1% for depression and 17–33% for anxiety in patients with BC, while in this study, prevalence rates for severe depression, anxiety, and stress in EBC group were 10%, 8%, and 10%, respectively. [4,7,52]. Differences in results among studies were mainly attributable to the heterogeneity of study designs, BC disease stage (early/metastatic disease), time since diagnosis, and methods of distress evaluation [52]. Meanwhile, similar to our findings, recent studies did not detect significantly higher levels of depression or anxiety in patients with EBC [53,54]. A retrospective analysis of patients newly diagnosed with EBC found low self-reported scores for depression and anxiety (no/minimal depression 81.5% and no/minimal anxiety 63.5%, respectively) [53], while another prospective study found no significant depression in EBC patients before treatment initiation [54]. There are numerous potential reasons for low distress levels in patients with EBC. Firstly, modern cancer therapies contribute to high overall survival rates and have relatively favorable side effects, resulting in better QoL, which has become one of the key factors in the decision-making process in prescribing EBC therapy [5,55]. Furthermore, effective supportive therapies prevent common side effects, including nausea, vomiting, loss of appetite, or febrile neutropenia, contributing to the QoL even more [5]. Also, psycho-oncology and the psychological support of patients with BC have been widely introduced and adopted as important parts of treatment, which in turn, alleviate symptoms of distress and strengthen resilience [56]. In addition, telecommunications, social networking, and telemedicine facilitate patient access to their peers, support groups, and medical staff, with additional soothing effects [37,57,58]. On the whole, significant improvements in the treatment of EBC with superior overall survival and better QoL, may lead to reduced distress levels among the EBC patients confronting the diagnosis.

Perception of health compared to 1 year prior differed significantly between the EBC and control groups. Considering the four domains of physical health, there were no differences between groups. This was expected, since the survey was conducted before EBC treatment commenced; accordingly, no difference regarding physical health (physical functioning, bodily pain, role limitations due to physical health problems, and role limitations due to personal or emotional problems) was found between groups. Patients with EBC had similar results on emotional health domains of the SF-36 scale (general mental health, social functioning, energy/fatigue, and general health perception) as controls, which is consistent with the DASS-21 scale results in this study.

This study showed the EBC group had significantly higher psychological resilience levels than controls. Few studies have addressed psychological resilience in patients newly diagnosed with EBC and matched controls [24,35]. Markovitz et al. found no significant difference in resilience between patients with EBC and controls [35]. However, the controls were significantly younger and had higher education levels, which is known to influence resilience levels [8,20,23]. Zhou et al. compared resilience in patients newly diagnosed with BC and normative control data. Their BC group had significantly lower scores of resilience when compared to control. On the other hand, they included early and metastatic BC patients, most of whom had started treatment, which could explain the differences compared with our findings [24]. Similar to our study, Mohlin et al. measured resilience levels in newly diagnosed EBC patients using CD-RISK 25 scale, but without a control group, and their EBC group showed a slight but significant decline in resilience levels in one year follow up [29]. According to the results of this research, it is hard to get an unequivocal answer to the question of how being diagnosed with early breast cancer impacts resilience.

The results of higher psychological resilience could be explained in light of particular features of exposure to the diagnosis of newly diagnosed EBC as an adverse life event. Several related studies on resilience have shown that experiencing challenging situations throughout life helps to build resilience [17,18,20]. Research of lifetime adversities and resilience in a national sample (N = 2.398) found that high/long-time and minor/no adverse events were associated with lower resilience, while people experiencing moderate/time-limited adversities were more resilient [18]. Therefore, moderate exposure to stressors can have “a positive effect on resilience when the exposure is limited, with an opportunity for recovery” [18]. Dooley et al. also found that previous exposure to moderate adverse events led to higher resilience relative to no or major adverse exposures and that acute, but not chronic stress, had a positive impact on resilience [17]. Considering these findings EBC diagnosis could represent an acute, moderate, and time-limited adverse event, with a positive effect on resilience. As both groups were well-balanced across sociodemographic factors and previous traumatic and threatening experiences, we may conclude that facing EBC diagnosis might have enhanced/strengthened resilience in EBC group.

Several pathways could potentially lead to higher resilience in the EBC group. Diagnostic procedures for BC (core needle biopsy, mammography, ultrasound of the breasts and axillary lymph nodes, and/or breast magnetic resonance imaging) typically take 4–6 weeks and represent a significant burden, which activates coping mechanisms and adaptive adjustments even before receiving a BC diagnosis [59,60]. Furthermore, only patients with EBC who were treated with curative intent were recruited in this study. Curative treatment is associated with higher resilience levels when compared with palliative treatment for metastatic disease [21,61]. Additionally, >90% of our EBC patients had stage I and II EBC, which is associated with 5-year survival rates of 96%. Therefore, EBC diagnosis could be classified as an adverse event with “limited exposure and opportunity to recover”, which is known to enhance resilience [17,18,61]. The CD-RISC 25 scale, used in this research, measures the dynamic, variable component of resilience and captures changes in psychological resilience 4 weeks after an intervention/exposure to adversity [38,62,63]. Hence, the diagnostic period needed to verify EBC diagnosis may induce activation of coping mechanisms and promote beneficial interaction with environmental resources, leading to higher resilience, as shown in our study. Although we compared EBC and healthy controls, it is important to note we collected the data after the EBC participants had been diagnosed with breast cancer. Since we do not have the baseline, pre-diagnosis data for distress, psychologic resilience, and the perception of health, our results should be interpreted with caution, as they do not allow causal interpretation.

### Limitations and Future Directions

This study has several limitations. First, personality-related variables, social context, coping strategies, and economic resources, which could determine a response to adverse events [8,11,34], were not fully explored due to our study design. Second, the healthy control group could introduce selection bias. In this research, the response rate for control group was 52%, and we lack the data about possible difference in demographics between women who refused to participate and those who participated in the study as controls. Despite that, the EBC group and healthy controls were very well balanced regarding socio-demographical characteristics, chronic illnesses, and previous exposure to stress and traumatic events. Third, the single-center study design could represent a limitation of generalizability since cancer care organization, trust in treatment, and quality of health care may be reflected in distress and resilience levels [29]. Further, we had a small sample size, and therefore the results should be interpreted with caution. Finally, we only included EBC patients up to 70 years of age. BC incidence rates are highest at the age 65–69 and around 30% of cases are diagnosed at older ages [64]. Consequently, our EBC group does not completely represent patients with BC, and our results cannot be generalized across the EBC population.

## 5. Conclusions

Psychological resilience in patients with cancer has recently become the focus of interest in numerous studies. To the best of our knowledge, this is the only study comparing resilience levels in newly diagnosed EBC patients and healthy controls, and we found EBC patients to be more resilient, suggesting that being diagnosed with early breast cancer might induce effective adaptation to acute stress, leading to higher resilience levels. Additional longitudinal research should explore whether higher resilience levels among EBC patients could also lead to better treatment and survival outcomes.

## Figures and Tables

**Table 1:** Comparison of demographic characteristics between participants in the breast cancer (*N* = 50) and control (*N* = 67) groups (*χ*^2^ test).

		Breast Cancer Group N (%)	Control Group N (%)	*χ*^2^ (*df*)	*p*
Marital status	Unmarried	2 (4.0)	3 (4.5)	0.697 (3)	0.874
	Divorced	5 (10.0)	10 (14.9)		
	Partnership/married	36 (72.0)	46 (68.7)		
	Widow	7 (14.0)	8 (11.9)		
Dependent children	No children	3 (6.0)	7 (10.4)	0.729 (2)	0.695
	Yes	12 (24.0)	15 (22.4)		
	No	35 (70.0)	45 (67.2)		
Educational level	Primary school	2 (4.0)	3 (4.5)	0.029 (2)	0.985
	Secondary school	32 (64.0)	42 (62.7)		
	University/PhD	16 (24.0)	22 (31.3)		
Working status	Employed	25 (50.0)	38 (56.7)	0.943 (2)	0.624
	Unemployed	3 (6.0)	2 (3.0)		
	Retired	22 (44.0)	27 (40.3)		
Monthly income	Insufficient	10 (20.0)	21 (31.3)	6.290 (3)	0.098
	Sufficient	29 (58.0)	41 (61.2)		
	More than sufficient	10 (20,0)	5 (7.5)		
	Much more than sufficient	1 (2.0)	0 (0.0)		
Comorbidities	Yes	21 (42.0)	24 (35.8)	1.940 (2)	0.379
	No	28 (56.0)	43 (64.2)		
Previous trauma according to LEC	Yes	24 (48.0)	30 (44.8)	0.184 (2)	0.912
	No	25 (50.0)	36 (53.7)		
Previous stress according to LTE	Yes	13 (26.0)	21 (31.3)	0.557 (2)	0.757
	No	36 (72.0)	44 (65.7)		
Breast cancer stage	I	34 (68.0)			
	II	13 (26.0)			
	III	3 (6.0)			

LTE, List of Threatening Experiences; LEC, Life Events Checklist.

**Table 2:** Descriptive statistics and comparison of participants in the breast cancer (N = 50) and control (N = 67) groups for DASS-21, SF-36, and CD-RISC-25 subscale scores (*t*-test) when controlling for age.

Scale	Subscale	Breast Cancer Group *M* (*SD*)	Control Group *M* (*SD*)	*t* (*df*)	*p*
DASS-21	Depression	7.2 (7.78)	5.0 (6.24)	2.971 (115)	0.087
	Anxiety	5.0 (7.59)	3.1 (5.49)	2.447 (115)	0.121
	Stress	10.1 (10.12)	8.4 (8.02)	1.081 (115)	0.301
SF-36	Physical functioning	74.2 (23.89)	80.8 (20.82)	2.304 (115)	0.132
	Role limitations due to physical health problems	62.5 (39.20)	70.5 (39.87)	0.969 (115)	0.327
	Role limitations due to emotional problems	69.3 (36.78)	81.1 (33.45)	2.967 (115)	0.088
	Energy/fatigue	59.8 (19.14)	56.9 (16.40)	−0.806 (115)	0.371
	General mental health	67.9 (17.17)	68.2 (17.13)	0.001 (115)	0.970
	Social functioning	67.8 (22.88)	75.0 (22.82)	−1.394 (115)	0.240
	Bodily pain	72.6 (24.87)	76.0 (21.57)	−0.473 (115)	0.493
	General health perception	63.6 (15.19)	66.2 (15.89)	−0.554 (115)	0.458
	Perception of health compared to 1 year prior	33.5 (22.37)	48.0 (18.44)	−14.407 ** (115)	0.000
CD-RISK25		77.8 (13.64)	71.78 (12.19)	−6.249 * (115)	0.014

Notes: * *p* < 0.05 ** *p* < 0.01. DASS-21, Depression Anxiety Stress Scale; SF-36, SF-36-Item Health Survey 1.0; CD-RISK25, Connor–Davidson Resilience Scale.

## Data Availability

The data presented in this study are available on request from the corresponding author.
